# Taylor’s Medical Jurisprudence

**Published:** 1845-03

**Authors:** 


### TAYLOR'S MEDICAL JURISPRUDENCE.

From the same publishers we have received a copy of Taylor's Medical Jurisprudence, with notes and additions by Dr. Griffith. The size of this volume renders it convenient, and altogether it appears to us adapted to its purpose—that of forming a guide to the medical jurist. It ought to be studied, and we can venture to say that few books in the same compass contain a greater amount of instructive and interesting matter. We shall have more to say of it hereafter. Y.

